# Advancements in Nanocarrier Systems for Nose-to-Brain Drug Delivery

**DOI:** 10.3390/ph18050615

**Published:** 2025-04-23

**Authors:** Thi-Thao-Linh Nguyen, Van-An Duong

**Affiliations:** 1 Institute of Pharmaceutical Education and Research, Binh Duong University, Thu Dau Mot City 820000, Binh Duong, Vietnam; nttlinh@bdu.edu.vn; 2 The Institute of Molecular Medicine, The University of Texas Health Science Center at Houston, Houston, TX 77030, USA

**Keywords:** nose-to-brain, nanoparticles, micelles, emulsions, liposomes, SLNs, NLCs, blood–brain barrier, brain, bioavailability

## Abstract

In recent decades, nose-to-brain drug delivery has shown effectiveness in treating many central nervous system diseases. Intranasally administered drugs can be delivered to the brain through the olfactory and trigeminal pathways that bypass the blood–brain barrier. However, nose-to-brain drug delivery is challenging due to the inadequate nasal mucosa absorption of drugs and the short retention time of the intranasal formulations. These problems can be minimized through the use of nano-drug delivery systems, such as micelles, polymeric nanoparticles, nanoemulsions, liposomes, solid lipid nanoparticles, and nanostructured lipid carriers. They can enhance the drug’s bioavailability in the brain via increases in drug solubility, permeation, and stability. Nose-to-brain nano-drug delivery systems have been evaluated in vivo by a number of research groups. This review aims to provide an overview of nose-to-brain delivery and recent advances in the development of nano-drug delivery systems for delivering drugs from the nose to the brain to improve the treatment of some central nervous system diseases.

## 1. Introduction

Intranasal (IN) administration has emerged as a widely utilized, non-invasive approach for drug delivery, offering the potential for both local effects within the nasal cavity and systemic circulation. Drugs delivered through this route can either remain localized in the nasal cavity for targeted treatment or be absorbed through the nasal mucosa, entering the bloodstream for broader systemic effects [1]. Over the past decade, this method has gained considerable attention as a promising route for delivering drugs directly to the brain, taking advantage of the anatomical connection between the nasal cavity and the central nervous system [2,3,4,5]. The direct nose-to-brain transport via the olfactory and trigeminal nerve pathways offers a rapid and efficient method for delivering drugs to the brain, making it a valuable approach for managing various central nervous system diseases and psychiatric disorders [6,7]. Unlike oral administration, IN delivery bypasses gastrointestinal and hepatic metabolism, improving drug bioavailability. Additionally, IN administration minimizes drug accumulation in non-target organs, thereby reducing systemic side effects and enhancing the overall safety profile of treatments [8,9,10]. Compared to parenteral administration, IN delivery bypasses the blood–brain barrier (BBB), resulting in higher drug accumulation in the brain, increased bioavailability, and enhanced therapeutic effects [11]. Additionally, direct nose-to-brain delivery offers a rapid onset of drug action, which is crucial for managing acute conditions. IN administration also improves patient compliance (as it is non-invasive) and allows self-administration [12]. However, IN drug delivery faces challenges such as limited dosing volume, rapid mucociliary clearance, enzymatic degradation, and the poor permeability of certain drugs, along with potential nasal irritation and variability in absorption due to nasal conditions [13,14,15]. Addressing these requires careful formulation design, including pH compatibility, the use of mucoadhesive agents, and strategies to enhance drug stability and absorption [16].

Nanotechnology has significantly impacted pharmaceutical sciences, particularly in the development of nanosized drug delivery systems over the past few decades. These nanoscale systems, typically composed of particles smaller than 1000 nm, offer numerous benefits, including a high surface area, enhanced drug solubility, improved absorption, and greater stability [17,18]. Common nanocarriers for drug delivery include micelles, liposomes, nanoemulsions, polymeric nanoparticles (NPs), solid lipid nanoparticles (SLNs), and nanostructured lipid carriers (NLCs) [19,20,21,22]. These systems have been increasingly employed for nose-to-brain drug delivery as they have the potential to overcome some challenges associated with IN administration. By extending the residence time in the nasal cavity, enhancing solubility and absorption, and increasing drug stability, nano-drug delivery systems significantly improve drug accumulation in the brain and boost therapeutic efficacy [23,24]. Additionally, some of these nanosystems can provide controlled or sustained drug release, further optimizing drug delivery and reducing the frequency of administration. Furthermore, nanocarriers can be tailored to target specific brain regions or receptors, offering more precise treatment for neurological disorders [25,26].

This review focuses on nano-drug delivery systems to deliver drugs from the nose to the brain. We provide an overview of nose-to-brain pathways and summarize recent advances in nose-to-brain nano-drug delivery systems for improving the treatment of some central nervous system (CNS) diseases.

## 2. Structural Features Relevant to Drug Delivery and Pathways for Drug Transport to the Brain

**Figure 1:**
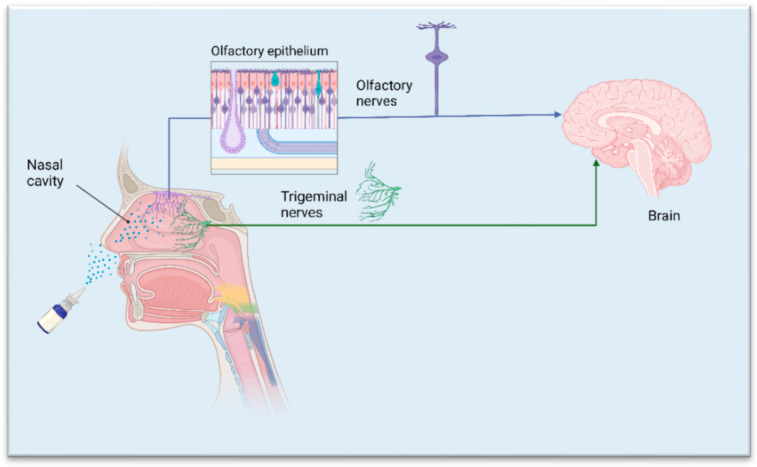
The olfactory and trigeminal nerve pathways are in direct nose-to-brain drug transport. Reprinted from [11] under the Creative Commons Attribution (CC BY) license (https://creativecommons.org/licenses/by/4.0/, accessed on 1 April 2025). The original figure was created with BioRender.com.

Nose-to-brain delivery via IN administration is based on several unique structural features of the nasal cavity and associated neural pathways, which are critical for effective drug delivery to the brain [27,28,29]. The nasal cavity is lined with a highly vascularized mucosal epithelium, which facilitates the rapid absorption of drugs. Two primary neural pathways, the olfactory and trigeminal nerves, play crucial roles in direct nose-to-brain drug transport (Figure 1) [30,31]. The olfactory region, located in the upper part of the nasal cavity, provides a direct connection to the brain through the olfactory nerve [31]. Drugs can be transported extraneuronally along olfactory neurons in a short time (<30 min). This is the primary route for drugs to enter the brain directly. In addition, drugs can be endocytosed by olfactory neurons, transported to the olfactory bulb, and distributed to different brain regions. This process takes longer than the extraneuronal route (several hours or days) [30,32]. A small amount of drugs is transported by supporting cells via endocytosis or crossing the epithelium’s tight junctions. The surface area of the olfactory epithelium is about 5 cm^2^ [33], which allows for efficient drug absorption, though its limited anatomical location in the upper nasal cavity can pose a challenge for drug deposition. Some nano-drug delivery systems can enhance drug retention in this area. The trigeminal nerve, which innervates both the respiratory and olfactory regions of the nasal cavity, also provides a route for drugs to bypass the BBB [30,34]. It extends into the CNS through branches that connect to the brainstem, enabling drug transport to the brain. 

Following IN administration, drugs can enter the brain through the indirect pathway. Drugs distributed in the respiratory region will be absorbed by the respiratory epithelia, enter the systemic circulation, cross the BBB, and then reach the brain. This pathway is suitable for many lipophilic drugs with low molecular weights and high permeability. IN administered drugs can also be lost to the lungs and gastrointestinal tract, where they are absorbed into the bloodstream [30,35]. The drug may enter the brain by crossing the BBB from systemic circulation. However, many efflux pumps in the BBB play a critical role in preventing drugs from entering the brain, making this indirect pathway less remarkable [36,37]. In addition, BBB restricts drug entry through its specialized physical structure, including tightly connected endothelial cells with complex tight junctions, the absence of fenestrations, and low rates of transcytosis [38].

## 3. Factors Affecting Nasal Absorption for Nose-to-Brain Delivery

### 3.1. Properties of Drugs

The molecular weight of a drug is crucial in influencing its absorption through the nasal mucosa. Generally, drugs with a molecular weight below 1000 Da can permeate the nasal epithelium effectively through cell–cell tight junctions [38,39]. Larger molecules may have difficulties crossing the nasal barrier, limiting their effectiveness in nose-to-brain delivery.

The lipophilicity of a drug significantly affects its nasal absorption. Lipophilic drugs tend to diffuse across the nasal mucosa more readily via the transcellular route [39]. Enhanced lipophilicity can facilitate drug penetration and improve absorption rates. Conversely, highly hydrophilic drugs may have reduced absorption due to poor permeability through the nasal membrane [40].

The drug’s solubility in the nasal cavity’s fluids impacts its bioavailability [41]. Poorly soluble drugs may not dissolve adequately in the nasal secretions, leading to insufficient absorption. Thus, improving drug solubility in the formulation vehicle can increase its availability and nasal absorption.

The stability of a drug within the nasal formulation is essential for effective delivery. Drugs that degrade or undergo significant chemical changes within the nasal environment may exhibit reduced therapeutic efficacy [6]. Stable formulations ensure the drug remains active and effective throughout the delivery process [42].

The pH of the nasal formulation can influence drug stability and absorption. The nasal mucosa typically has a pH range from 4.5 to 6.5 [43,44]. Formulations should be adjusted to match this pH range to minimize irritation and enhance drug absorption. Deviations from this pH range may lead to drug instability or mucosal damage. The pKa of a drug is the pH at which the ionized and unionized drug concentrations are equal. The unionized form of a drug can cross the nasal mucosa most easily due to its lipophilicity. The difference between the nasal cavity’s pH and the drug’s pKa determines how much of the drug is unionized. At a certain pH of the nasal mucosa (4.5–6.5), an acidic drug with a pKa < 4 is mostly unionized, favoring drug absorption. Reversely, a basic drug with a pKa > 8 is mainly ionized, and thus, the absorption is reduced. In this case, we can use buffers to increase the formulation pH closer to 8, thereby increasing the unionized drug to improve drug absorption. However, formulations with pH < 4.5 or >7.0 may induce irritation due to the mucosal inflammation. The solutes in buffers may increase osmolarity, and adjustment is required to maintain the formulation osmolarity near isotonic (ideally 280–310 mOsm/L) [45].

### 3.2. Formulation Properties

With a sub-micron size, nanoparticles can permeate the lipid membranes or cross the tight junctions of the nasal epithelium [11,46,47]. Larger particles may be trapped in the anterior part of the nasal cavity, reducing the effectiveness of the brain delivery. When these larger particles reach the mucosal tissue, they need to release the drug for absorption by passive diffusion [48]. 

Permeation enhancers are additives in nasal formulations designed to improve drug absorption by temporarily modifying the nasal mucosa’s permeability. Examples include polysorbate 80, dodecyl maltoside, tetradecyl maltoside, methyl-β-cyclodextrin, and chitosan (CS) [49,50]. These enhancers can disrupt tight junctions or alter mucosal structure to facilitate drug uptake [51]. However, their use must be carefully controlled to avoid mucosal damage or irritation.

The viscosity of the nasal formulation affects its retention time and distribution in the nasal cavity. Higher viscosity formulations may remain in the nasal cavity longer, allowing for prolonged mucous contact and enhanced absorption [52,53]. Conversely, formulations that are too viscous may cause discomfort or difficulty in administration. The high viscosity also reduces the drug release and permeation. Polymers such as carboxymethylcellulose, carbomers, and poloxamers are usually included in an intranasal formulation to increase its viscosity [54].

Osmolarity is another critical factor for nasal formulations. The osmolarity of the nasal formulation can impact mucosal hydration and drug absorption [55]. Formulations that are isotonic with nasal secretions (i.e., those that have an osmolality range between 280 and 310 mOsm/L) are generally better tolerated and less likely to cause irritation. A wider osmolarity range (200–600 mOsm/L) can be used in formulations without affecting the integrity of the nasal mucosa [56]. Hypertonic or hypotonic solutions may lead to discomfort or reduced absorption efficiency [57,58]. Hypotonic solutions (<280 mOsm/L) cause water to move into cells, potentially leading to swelling and discomfort. Hypertonic solutions (>310 mOsm/L) draw water out of cells, leading to shrinkage, dryness, or irritation.

Nasal deposition is a critical factor in determining how effectively a drug is absorbed. The type of delivery device used to administer the nasal formulation can influence the formulation deposition in the nasal cavity and alter the drug absorption. Nasal sprays, pumps, or nebulizers can affect the distribution and deposition of the drug within the nasal cavity [54]. Dry powder insufflators (DPIs) and pressurized metered-dose inhalers (pMDIs) are usually used to deliver powder. The unidose (UDS) powder nasal spray system can quickly and efficiently deliver a single precise dose [59]. For liquid, the traditional nasal drops and sprays often deposit drugs in the anterior nasal cavity or turbinate regions, where absorption is slower, and mucociliary clearance quickly sweeps drugs toward the throat. The new device design ensures uniform distribution and effective delivery to the target regions. Nebulizers can generate aerosolized droplets for deep penetration into the nasal cavity. Modern clinical nebulizers include pneumatic jet nebulizers, ultrasonic nebulizers, and vibrating mesh nebulizers [60]. The TriVair™ device can deliver drugs deep into the nasal cavity and reach the olfactory nerve, which enables a rapid onset of action and effective brain delivery [61]. Kurve’s ViaNase device is an electronic atomizer that creates a vortex of nebulized particles, usually used for monoclonal antibodies and larger peptides. It enhances the olfactory deposition of drugs for nose-to-brain delivery. Precision olfactory delivery (POD) can deliver drugs specifically to the upper nasal space [62]. The Sipnose device employs a pressurized system using compressed air to generate an aerosol with a precisely controlled narrow plume. This design enhances targeted drug delivery to the olfactory epithelium, improving nasal absorption efficiency [63].

### 3.3. Nasal Cavity Conditions

The integrity of the nasal mucosa plays a crucial role in drug absorption. Damage or inflammation of the mucosa, such as from chronic rhinitis, sinusitis, or nasal trauma, can affect drug absorption. Healthy, intact mucosal surfaces facilitate better drug uptake, while compromised mucosa can lead to reduced efficacy or inconsistent drug delivery [64]. Nasal temperature can influence drug absorption. In humans, the temperature within the nasal cavity typically ranges from approximately 31 °C to 34 °C under normal physiological conditions [65]. This range is influenced by factors such as respiratory airflow, mucosal blood flow, and ambient temperature. While the body generally maintains homeostasis, exposure to extreme environmental conditions, such as very cold or hot air, can transiently affect the local nasal temperature. However, due to the nasal mucosa’s efficient thermoregulatory mechanisms, these fluctuations are usually moderate and short-lived. Variations in temperature can affect the viscosity and solubility of nasal formulations [64]. For example, colder temperatures may lead to increased viscosity, affecting drug release and absorption. Optimal drug delivery systems should account for potential temperature variations in the nasal cavity. Considering the environmental temperature and humidity can help in designing formulations that maintain efficacy under varying environmental conditions. Nasal blood flow can affect the rate of drug absorption and its subsequent transport to the brain. Drug uptake can be increased when blood flow increases and vice versa. Vasoconstriction can reduce drug absorption by limiting blood flow to the nasal mucosa, while vasodilation can enhance absorption by increasing blood flow. These effects can be influenced by physiological responses or pharmacological agents used in the nasal formulation [66].

Mucociliary clearance in the nasal cavity is an active defense mechanism for protecting the body against pathogens [56]. However, it also limits drug residence time and reduces drug absorption. Some mucoadhesive agents are incorporated into a nose-to-brain formulation to prolong drug contact with the nasal epithelium [58]. The humidity of the nasal cavity affects drug dissolution and absorption [40]. The nasal mucosa is normally well-hydrated, with in vivo relative humidity ranging from 95% to nearly 100% under physiological conditions [65]. This high humidity supports the formation of a thin aqueous layer on the epithelial surface, which facilitates the dissolution of drug particles before absorption. Low humidity can lead to thickened mucus, which may hinder drug penetration and absorption. Conversely, excessively high humidity can alter the drug’s solubility and stability. However, for drugs administered as aqueous solutions via nasal sprays, which are the predominant dosage form for nose-to-brain delivery, humidity has a minimal direct impact on dissolution because the drug is already in a dissolved state upon administration. Humidity is critical for dry powder or suspension formulations.

Increased mucus production, as seen in conditions like allergies or respiratory infections, can dilute or trap nasal formulations, reducing drug absorption. High mucus production can also impede the delivery device’s effectiveness. Formulations may need to be designed to cope with or minimize the impact of excess mucus [67].

### 3.4. Roles of Nano-Drug Delivery Systems in Nose-to-Brain Delivery

Nano-drug delivery systems have been used to enhance the efficacy and targeting of therapeutic agents. In nose-to-brain delivery, these nanocarrier systems offer several key advantages and functionalities that are crucial for overcoming the barriers associated with this route of administration.

#### 3.4.1. Enhanced Drug Retention and Penetration

Nanocarriers can effectively penetrate the nasal mucosa due to their small size. NPs with a size below 200 nm are considered optimal for effective transport via the olfactory and trigeminal nerve pathways, while those below 100 nm can minimize mucociliary clearance and facilitate nerve-mediated transport to the brain [28,68]. This enhances the brain bioavailability of the drug. The lipophilicity of some lipid-based nanocarriers, such as emulsions, liposomes, SLNs, and NLCs, can increase the partition of NPs into the lipid bilayer of the nasal epithelial cell membrane [69]. Nanoparticles with adequate lipophilicity can readily pass through the intercellular spaces between olfactory cells [70]. Additionally, incorporating surfactants such as Tween 20, Tween 80, and sodium lauryl sulfate can increase drug permeation by solubilizing membrane lipids, altering membrane fluidity, and enhancing transcellular transport. These surfactants can also open tight junctions between epithelial cells, thereby enhancing drug permeability [71]. Nanocarriers prepared with poloxamer can decrease mucus viscosity and elasticity, thereby increasing their transcellular transport [72].

Incorporating drugs into nanocarriers can enhance drug retention within the nasal cavity [73]. The retention time can be further improved after embedding these nanocarriers into gels or coating them with suitable materials. Commonly used agents for gel preparation include Poloxamer 407, Poloxamer 188, methylcellulose, and hydroxypropyl methylcellulose. Certain nanocarriers are designed to adhere to the mucosal surface through surface modification, which extends their residence time in the nasal cavity. This mucoadhesive property helps maintain prolonged contact with the mucosa and enhances drug absorption [74,75]. Modifying the surface of NPs can adjust their charge, which enhances mucoadhesion and drug absorption. For instance, positively charged NPs can adhere more effectively to negatively charged mucosal surfaces, which enhances mucoadhesion and extends their residence time [76]. Altering the surface charge of nanocarriers from negative to positive strengthens the electrostatic attraction to mucus [77]. For example, coating ferulic acid-loaded SLNs with CS resulted in an increased mucoadhesive strength and drug accumulation in the brain as well as enhanced cognitive function in Alzheimer’s disease (AD)-induced rats [78]. Other mucoadhesive polymers, such as trimethyl CS [76] and glycol CS [79], can also extend the residence times of NPs in the nasal cavity. Nanocarriers coated with polyethylene glycol 25 stearate can inhibit P-glycoprotein efflux, presenting at cerebrovascular endothelial cell membranes, thereby increasing brain drug concentration [80].

Some components of the nanocarriers may act as absorption promoters, which increase the drug transport by modulating membrane permeability, opening tight junctions, or facilitating transcytosis. Surfactants (e.g., polysorbates, bile salts), fatty acids (e.g., oleic acid), chitosan derivatives, and cyclodextrins help increase drug solubility and permeability. Other agents like disodium ethylenediaminetetraacetate (EDTA) and Solutol^®^ HS15 can act as tight junction modulators, transiently disrupting tight junctions to enhance paracellular transport [81].

#### 3.4.2. Enhanced Stability and Reduced Side Effects

Nano-drug delivery systems can encapsulate and protect drugs that are sensitive to degradation. By shielding these drugs from environmental factors such as pH changes, oxidative conditions, and enzymatic degradation [70], nanocarriers enhance the stability and efficacy of the therapeutic agents. Nanocarriers can maintain the physical and chemical stability of the drug during storage and administration, ensuring that the drug remains effective when it reaches the nasal mucosa.

By focusing drug delivery directly to the olfactory region and, subsequently, to the brain, nanocarriers reduce the amount of drug that enters the systemic circulation. This decreases the risk of systemic side effects and toxicity, improving the overall safety profile of the treatment [69].

## 4. Evaluation of Intranasal Formulations for Nose-to-Brain Delivery

To assess the efficiency, safety, and targeting capability of IN formulations for nose-to-brain delivery, various in vitro, ex vivo, and in vivo evaluation methods are employed. These approaches help in understanding drug permeability, mucosal interaction, pharmacokinetics, and brain-targeting efficiency.

Particle size, zeta potential (ZP), and polydispersity index (PDI) are used to determine the stability and nasal mucosa penetration potential of drug nanocarriers. An analysis of pH and osmolarity can ensure formulation compatibility with the nasal mucosa to prevent irritation. Viscosity and gelation studies evaluate the formulation’s ability to adhere to the nasal mucosa for prolonged retention [26,82]. Formulations are also assessed for pH, which must align with nasal mucosa tolerance (4.5–6.5) to avoid irritation while optimizing the drug’s ionization state based on its pKa. Osmolality should fall within 200–600 mOsm/L (ideally, 280 and 310 mOsm/L, isotonic) to prevent mucosal swelling or shrinkage, which could impair ciliary function or drug transport to the brain.

In vitro drug release studies are useful for understanding drug release kinetics. These studies can be conducted using dialysis membranes or Franz diffusion cells. In vitro permeability studies can be assessed using cell culture models to mimic nasal epithelial barriers for permeability assessment [83]. RPMI 2650 is a human nasal epithelial cell line derived from the septum. It is widely used due to its ability to form tight junctions and mucus-secreting properties. Primary olfactory epithelial cells are more physiologically relevant. However, they are harder to culture and less reproducible [84]. Moreover, other cell lines can be used to mimic epithelial barriers for permeability assessment, such as Calu-3, a human airway epithelial cell line, and Caco-2, a human colon epithelial cell line. However, they are not the best choice for evaluating nose-to-brain delivery [85].

Cytotoxicity assays (e.g., MTT, LDH release assay) are carried out to assess nasal epithelial cell viability upon exposure to formulations [86]. In vitro mucoadhesion studies can be carried out by measuring the adhesion strength of formulations using mucin-binding or rheological methods [87,88].

Deposition performance, including spray performance and nasal cast deposition, is a critical evaluation component for intranasal formulations aimed at nose-to-brain delivery, as it determines how effectively the drug reaches the olfactory region for direct CNS access [89]. The spray performance examines the formulation’s delivery characteristics, such as the plume geometry produced by advanced devices like the Aptar UDS nasal spray system or POD. It also considers droplet or particle size distribution, spray velocity, and dose uniformity to ensure that each actuation delivers a consistent therapeutic amount. The nasal cast deposition uses 3D-printed models based on human anatomy. The goal is to achieve significant deposition in the olfactory epithelium, ideally exceeding 20–30% of the dose [3].

Ex vivo evaluations use excised nasal mucosa from animals such as sheep, pigs, or goats to better replicate in vivo conditions. These studies measure drug permeability using Franz diffusion cells and Ussing chambers, while histological analyses assess potential tissue damage [82]. These evaluations provide critical insights into drug transport across the nasal mucosa, enabling researchers to refine formulations for optimal brain targeting while minimizing adverse effects on the nasal epithelium.

In vivo studies are conducted in animal models, including rodents and larger animals like rabbits and monkeys, to evaluate pharmacokinetics, biodistribution, and therapeutic efficacy. Imaging techniques are used to track drug distribution and penetration of the blood-brain barrier [90]. Depending on the disease, different pharmacodynamic studies are employed to evaluate therapeutic outcomes in neurodegenerative disease models. Toxicity and safety studies are also carried out, including histopathological examination of the nasal mucosa and brain tissues, inflammatory response analysis through cytokine profiling, and nasal irritation assessments via nasal lavage analysis [91]. Although rodent models are widely used in intranasal drug delivery studies due to their ease of handling and well-established protocols, they present notable limitations. The requirement for anesthesia during administration can alter nasal physiology and drug absorption. Additionally, rodents possess a proportionally larger olfactory region than humans [92], which may lead to an overestimation of the nose-to-brain transportation. These anatomical and physiological differences highlight the importance of validating findings in larger animal models or employing advanced in vitro systems that mimic the human nasal environment better.

In pharmacokinetics studies, different parameters are used to evaluate the IN nanocarriers for brain targeting efficiency, such as AUC_brain_, C_max (brain)_, T_max (brain)_, mean residence time (MRT), drug targeting efficiency (DTE%), and drug transport percentage (DTP%) [30]. DTE% quantifies the extent to which a drug preferentially accumulates in the brain following IN administration compared to systemic circulation. It is calculated as the ratio between AUC_brain_/AUC_blood_ for the IN route and AUC_brain_/AUC_blood_ for the IV route. DTE% values can range from 0 to infinity. A higher DTE% suggests that the formulation successfully bypasses systemic circulation and delivers the drug directly to the brain, minimizing peripheral side effects.

DTP% represents the proportion of the drug that reaches the brain directly through the nose-to-brain pathway, bypassing systemic circulation. It is determined as the ratio between AUC_brain (direct)_ and AUC_brain (total)_ for IN nanocarriers. The AUC_brain (direct)_ is calculated as AUC_brain (total)_ − AUC_brain (indirect)_, in which the AUC_brain (indirect)_ represents the drug entering the brain from the bloodstream by crossing the BBB and can be determined using AUC_blood (IN)_ × AUC_brain (IV)_/AUC_blood (IV)_. The DTP% can also be calculated as 1–100/DTE% [37]. A high DTP% indicates effective transport via olfactory and trigeminal nerve pathways, confirming the formulation’s ability to enhance direct brain delivery.

## 5. Nanocarriers for Nose-to-Brain Delivery

In this section, we summarize recent advances in nose-to-brain delivery using nanocarrier systems, including micelles, polymeric nanoparticles, emulsions, liposomes, SLNs, and NLCs. 

### 5.1. Micelles

Micelles are nanoscale colloidal assemblies that form when amphiphilic molecules, typically surfactants or block copolymers, self-assemble in an aqueous phase [93]. These molecules have both hydrophilic and hydrophobic regions. In water, the hydrophobic tails of the amphiphilic molecules cluster together to create a nonpolar core, while the hydrophilic heads face outward, interacting with the surrounding water [94]. This arrangement results in a spherical structure that can encapsulate hydrophobic substances within its core, making micelles efficient carriers for poorly soluble drugs [95]. Micelles can enhance the solubility, bioavailability, and stability of hydrophobic drugs in drug delivery [96,97]. Their nano size allows for better drug absorption, extended circulation times, and potential for targeted delivery [94]. Additionally, micelles can be engineered for controlled and sustained release of drugs, thereby reducing dosing frequency and minimizing systemic side effects. 

Micelles have been used for nose-to-brain drug delivery in many studies. Encapsulating risperidone into micelles increased drug permeation across a cellulose membrane [98]. Similarly, dexamethasone-loaded micelles exhibited an increase in the aqueous solubility of the drug (14-fold) and enhanced permeability [99]. Other studies reported in vivo evaluations to demonstrate the brain-targeting efficacy of micelles. For example, baicalein was loaded into poly(ethylene glycol)-block-poly(D, L-lactide) (PEG-PLA) micelles, which exhibited 1.50-fold higher AUC_brain_ than the oral drug powder following the IN inhalation [100]. Similarly, rotigotine-loaded micelles showed an extended MRT (1.43-fold) of the drug in rat plasma after IN administration compared to the IV free drug solution [101]. The micelles were then loaded into poloxamer gel, which further increased the MRT (1.79-fold). Compared to the IV group, rotigotine distribution increased by 276.6%, 170.5%, 166.5%, and 184.4% in the olfactory bulb, cerebrum, cerebellum, and striatum, respectively. 

Clozapine is a drug used to treat schizophrenia. It has low brain distribution following oral administration due to its low solubility, poor dissolution rate, degradation in the gastrointestinal tract, and high hepatic first-pass metabolism. In a previous study, clozapine-loaded polymeric nanomicelles were prepared using Tetronic^®^ 904 and 701 (two hydrophobic poloxamines) and Synperonic^®^ PE/F127 (a hydrophilic poloxamer) [102]. The optimized formulation exhibited five-fold higher flux compared with the free drug suspension with no histological irritation in an ex vivo nasal permeation study. In vivo biodistribution in mice showed higher brain distribution for the IN micelles compared with the IV micelles. The DTE% value was 396.5%, suggesting improved brain delivery via the IN route. Furthermore, T_max_ in the brain for IN micelles was 30 min, significantly faster than that of the IV micelles (120 min), indicating the quick onset of the IN route. In another study, clozapine was loaded into soya phosphatidyl choline and sodium deoxycholate micelles for nose-to-brain delivery [103]. The micelles exhibited three-fold higher ex vivo permeation compared with the free drug. Intranasal administration of the formulation to mice increased brain uptake and exhibited a quick onset of 15 min.

Ibudilast was loaded in polydopamine-coated surfactin micelles for IN administration [104]. The formulation increased drug distribution to the mouse brain and exhibited positive outcomes in treating multiple sclerosis (anti-inflammation and neuroprotection). Lurasidone-loaded mixed micelles of Gelucire 44/14 and Poloxamer 407 were developed using the solvent evaporation method and optimized through a 3² factorial design [105]. The micelles exhibited a size of 175 nm and an entrapment efficiency of 97.8%. The hydrogel with Carbopol 940 demonstrated enhanced ex vivo permeation (79%). Histopathological studies confirmed the absence of nasociliary toxicity in sheep nasal mucosa. The administration of the mixed micelles led to a significant improvement in brain drug concentration, with a half-life of 19.1 h, a DTE% of 394%, and a DTP% of 74%, emphasizing its efficacy for nose-to-brain drug delivery. Olanzapine was loaded into polymeric micelles of Poloxamer 407, Pluronic P123, and D-α-tocopherol polyethylene glycol 1000 succinate (TPGS) [106]. In rats, the IN micelles increased brain distribution with DTE% of 535.9% and DTP% of 81.3%. It also improved anti-schizophrenia-related deficits as indicated by the paw test and open field test.

Besides this advancement, some studies have reported unsuccessful IN micelles. In a previous study, meloxicam-loaded Soluplus micelles increased the drug permeation across the culture model of the nasal mucosa barrier [107]. Following the IN administration to rats, the drug can be found in the brain, but with a low AUC_brain_ compared to AUC_plasma_ (only 0.65%). Thus, although the nasal administration of the micelles helped to deliver the drug to the brain, most of the drug entered the bloodstream. There is no control group (IV or IN free drug) to compare, making it difficult to conclude the role of micelles in delivery to the brain. In another study, clozapine-loaded binary mixed micelles showed reduced permeation across nasal mucosal tissues [108]. The study did not include vivo data. Therefore, it is insufficient to conclude whether the IN clozapinemicelles improve the brain delivery of the drug. 

Table 1 summarizes the major features of micelle-based formulations for nose-to-brain delivery in these studies.

### 5.2. Polymeric Nanoparticles

Polymeric NPs are a class of nanocarriers made from biodegradable and biocompatible polymers [110]. These NPs typically range in size from 10 to 1000 nm and have gained significant attention in drug delivery due to their ability to improve the bioavailability, stability, and targeted delivery of drugs [111]. The key advantage of polymeric NPs lies in their versatility and ability to be tailored for various drug delivery applications. Through surface modification and functionalization, polymeric NPs can achieve targeted drug delivery, reduce systemic side effects, and provide controlled or sustained release of therapeutic agents [112]. They can also protect sensitive drugs from degradation and ensure a longer circulation time in the body. Additionally, the biodegradable nature of the polymers used, such as poly(lactic-co-glycolic acid) (PLGA), ensures that the nanoparticles break down safely and are eventually eliminated from the body [113]. 

Polymeric NPs have been widely studied for the nose-to-brain delivery of various drugs. For example, edaravone was loaded in PLGA NPs to improve the drug’s bioavailability in the brain for treating amyotrophic lateral sclerosis. In mice, IN edaravone-NPs offered higher and more sustained brain uptake of the drug [114]. Meloxicam can be used to treat neurodegenerative disorders to improve anti-amnesic activity in the brain. In situ thermo-gelling systems of meloxicam–human serum albumin NPs were developed to increase drug absorption in the nasal mucosa. The gel exhibited a higher meloxicam permeation ex vivo [115].

Baicalin-loaded PEG-PLGA NPs were coated with RVG29 peptide to improve the bioavailability of neuroprotective agents [113]. In rats with ischemic brain injury, the IN baicalin-NPs alleviated the neurological dysfunction, reduced the cerebral infarction area, and relieved nerve trauma and swelling. In addition, treatment with IN baicalin-NPs decreased the levels of IL-1β, IL-6, and TNF-α in rat serum, reduced the levels of reactive oxygen species and malondialdehyde, and increased the levels of glutathione and superoxide dismutase in rat brains. Therefore, after IN administration, the baicalin-NPs can effectively deliver baicalin to the rat brain via the nose-to-brain route and exert neuroprotective effects against ischemic brain injury.

Vinpocetine (VIN) is used to improve cognitive function, memory, and cerebrovascular disorders. VIN-loaded CS NPs were prepared using the ionotropic gelation technique [116]. The optimized NPs were incorporated into Poloxamer 407 and Poloxamer 188 gel. The IN administration of the VIN-NP gel increased C_max_ and brain bioavailability in rats 2.2- and 1.7-fold, respectively, compared with the oral VIN tablets.

Astragaloside IV (ASI), a compound with anti-inflammatory, antioxidant, remyelination, and neuroprotective activity, was loaded into β-asarone-modified CS NPs to improve its poor brain delivery due to the poor permeability [117]. The ASI-β-asarone-CS-NPs showed a 1.52-fold higher drug uptake in 16HBE cells and a 2.49-fold higher level in mouse brains than ASI-CS-NPs after IN administration. In experimental autoimmune encephalomyelitis mice, the ASI-β-asarone-CS-NPs exhibited decreases in behavioral scores, inflammatory infiltration, and astrocyte/microglial activation. It also decreased demyelination and improved remyelination. This study used β-asarone to increase drug permeability across the nasal mucosa [118].

Piribedil (PBD) has efficacy in treating motor and non-motor symptoms of Parkinson’s disease. It was entrapped into lecithin-CS NPs (PBD-LCNs) and then loaded in a thermo-responsive in situ gel (PBD-LCN-ISG) to increase the direct nose-to-brain uptake and reduce rapid mucociliary clearance after intranasal administration [119]. The bioavailability of PBD in the brain after IN administration of the PBD-LCN-ISG was 6.4-fold higher than that of PBD suspension. The PBD-LCNs showed a DTP% value of 56%, which suggests an efficient direct nose-to-brain uptake, while that value for PBD suspension is 0.

Paclitaxel-loaded PLGA NPs were developed to improve their efficacy against glioblastoma multiforme by nose-to-brain delivery [120]. Arginyl-glycyl-aspartic tripeptide (RGD) was coated onto the NPs’ surface to further increase the effectiveness due to the cancer-targeting properties of the peptide. While the IV administration showed the majority of the drug deposited in periphery organs (liver, spleen, and kidney), the IN PTX NPs and RGD-PTX-NPs exhibited the majority of the drug deposited in the glioma region of the brain with the peak at 12 h. In rats implanted with C6 glioblastoma cells, the tumor volume of IN PTX-NPs and RGD-PTX-NPs groups reduced 44% and 72%, respectively, compared to the PTX-treated controls. Similarly, in mice implanted with U87MG, the tumor volume reductions were 56% and 75%, respectively [120].

Clonidine is a drug used for the treatment of hypertension. It was encapsulated into transferrin-coated PLGA NPs [121]. Transferrin was used to increase the uptake of the NPs due to the high expression of transferrin receptors in the nasal epithelium and the brain. As a result, transferrin-coated PLGA NPs exhibited higher uptake by Neuro-2a cells compared with the uncoated PLGA NPs (97% vs. 82%). In mice, the drug concentration in the brain at 1.5 h after IN administration of transferrin-coated PLGA NPs was 2.4-fold higher than that of the IN free drug, whereas the uncoated PLGA NPs increased only 1.7-fold. Treating mice with IN transferrin-coated PLGA NPs reduced the anxiety and depression of the animals and improved their ability to focus and pay attention to the assigned objective or task.

Paroxetine is a drug used to treat depression. It was incorporated into PLGA NPs, and their surface was modified with CS to increase the mucoadhesive properties and colloidal stability for nose-to-brain delivery [121]. The paroxetine CS-PLGA NPs exhibited higher drug concentration in the mouse brains after IN administration. Also, the mice showed improved behavior in the forced swimming test and locomotor activity test.

Duloxetine (DXH) is a psychiatric drug used for the management of major depressive disorder. However, DXH has low water solubility, acid instability, and high first-pass metabolism, resulting in its low oral bioavailability. DXH was loaded into PLGA-CS-NPs for nose-to-brain delivery to improve brain accumulation [122]. The NPs were optimized using the Box-Behnken design. The optimized DXH-PLGA-CS-NPs exhibited four-fold higher ex vivo permeation than the DXH solution. IN administration of the optimized DXH-PLGA-CS-NPs in rats resulted in higher C_max_ (3.33-fold), AUC (3.57-fold), t_1/2_ (1.76-fold), and MRT (1.43-fold) in the rat brain than the oral DXH solution. Pharmacodynamics studies using the force-swimming test, tail suspension test, sucrose preference test, open field test, and novelty-suppressed feeding showed better behaviors in rats administered with the optimal DXH-PLGA-CS-NPs.

Table 2 summarizes the major features of polymeric NPs for nose-to-brain delivery in these studies.

### 5.3. Lipid-Based Nanocarriers

Lipid-based nanocarriers include emulsions, liposomes, SLNs, and NLCs. Emulsions are heterogeneous systems formed by blending two immiscible liquids, usually oil and water, with one dispersed as fine droplets within the other [124]. To stabilize this mixture and prevent phase separation, emulsifying agents or surfactants are used. Emulsions are generally classified into oil-in-water (O/W) emulsions and water-in-oil (W/O) emulsions. Depending on their droplet size, emulsions can range from macroemulsions (with larger droplets) to nanoemulsions (NEs), which contain droplets typically smaller than 100 nm [2]. Emulsions are efficient carriers for both hydrophilic and hydrophobic drugs, enabling controlled release, enhanced stability, and targeted delivery. 

Naringenin-loaded NEs in situ gel was developed using Poloxamer-407 as a thermoresponsive polymer and CS as a mucoadhesive agent [125]. Following IN administration in rats, the NEs increased drug accumulation to the brain with DTE% of 1224% and DTP% of 99.5%. Treating with IN gel improved the locomotor activity and grip strength in rats with ischemic brain. In addition, the pretreatment of rats with the IN NEs gel could increase the levels of antioxidant enzymes, including superoxide dismutase, catalase, glutathione peroxidase, and glutathione reductase.

Aripiprazole nanoemulgels were developed for nose-to-brain delivery to improve the management of schizophrenia [126]. The NEs were optimized using the Box-Behnken statistical design. The optimized nanoemulgel increased ex vivo permeation through sheep mucous membranes about two-fold compared with the drug solution. The pharmacokinetic profile following IN administration of the optimized aripiprazole nanoemulgel showed higher C_max_ and AUC in the brain than the IV administration. The recalculated DTE% was 974%, and the DTP% was 89.73%, indicating direct nose-to-brain delivery. In addition, the cataleptic test in Wistar rats showed the non-existence of the extrapyramidal side effect of the nanoemulgel. The paw test and locomotor test indicated the antipsychotic activity of the nanoemulgel.

Topiramate (TPM) is a drug used for the management of epilepsy. It is a substrate of P-glycoprotein, which limits its BBB permeation to enter the brain. In a previous study, TPM was loaded into NEs for nose-to-brain delivery [127]. The optimal TPM NEs had a globule size of 4.73 nm. The pharmacodynamic study in rats showed that IN TPM NEs significantly decreased average seizure duration and increased the percentage of inhibition of seizures compared to oral TPM NEs, IV TPM, and oral TPM. In the pharmacokinetic study in rats, the TPM distributed in the brain 1 h after administration follows the order: oral TPM < IN TPM < oral TPM NEs < IN TPM NEs. The TPM distributed in the brain at 1 h for IN TPM NEs was 1.33-, 2.48-, and 3.22-fold higher than that for oral TPM NEs, IN TPM, and oral TPM, respectively. 

Tetrabenazine is a drug widely used for Huntington’s disease. The NEs of tetrabenazine were developed for nose-to-brain delivery to improve the treatment of hyperkinetic movement associated with Huntington’s disease [128]. The optimized tetrabenazine NEs showed 1.68-fold higher ex vivo permeation than the free drug. In rats, the IN tetrabenazine NEs exhibited higher C_max_ (4-fold) and AUC (6.07-fold) in the brain compared with the IV drug solution. Although the authors did not report DTE% and DTP%, we could calculate them from the AUC values. The DTE% of 1666% and DTP% of 94% indicate the efficient direct nose-to-brain delivery of tetrabenazine NEs following IN administration.

Liposomes are spherical vesicles containing one or more phospholipid bilayers, similar to the structure of biological membranes [129]. They can encapsulate hydrophilic drugs in their aqueous core and lipophilic drugs within the lipid bilayers, making them highly versatile drug carriers [130]. Liposomes are biocompatible, biodegradable, and non-toxic, which makes them ideal for delivering a wide range of drugs, including anticancer agents, vaccines, and gene therapies. They offer enhanced drug protection, prolonged circulation times, and targeted delivery to specific tissues, thereby reducing systemic side effects [131]. Liposomes have been widely used for nose-to-brain delivery [7]. For example, imatinib mesylate-loaded liposomes increased AUC_brain_ (7-fold) compared with the oral and IN free drug solutions in rats [132]. Hydroxy-α-sanshool, an anti-AD compound, was loaded into liposomes for nose-to-brain delivery via IN administration [133]. Following IN administration to mice, the hydroxy-α-sanshool liposomes increased AUC_plasma_ (1.7-fold) and AUC_brain_ (2.1-fold) compared with the free drug. Liposomes can be loaded into hydrogels to increase residence time in the nasal cavity. For example, a liposome in situ gel was developed using Poloxamer 407 and Poloxamer 188 to encapsulate an anti-AD compound (name unrevealed) with a high aqueous solubility (>10 mg/mL) and limited permeability [134]. The liposome in situ gel exhibited a gelation temperature of 32.6 °C and increased mucoadhesion compared with the blank gel.

Transfersomes are vesicular carriers designed to enhance drug delivery by penetrating biological barriers, including the skin and mucosal membranes. They are modified from liposomes by incorporating an edge activator, such as surfactants or bile salts, which provide high flexibility and adaptability [135]. Due to their deformability, transferosomes can squeeze through tight intercellular spaces, making them highly effective for transdermal, intranasal, and other non-invasive drug delivery routes. They improve drug bioavailability, enhance penetration efficiency, and facilitate the targeted delivery of both hydrophilic and lipophilic drugs [136]. Their application in nose-to-brain drug delivery holds promise for improving therapeutic outcomes in neurological disorders. For example, donepezil (DPZ), an FDA-approved cholinesterase inhibitor used for the management of AD, was encapsulated into hyaluronic acid-coated transfersomes (DPZ-HA-TFS) for nose-to-brain delivery to avoid the oral-correlated side effects of DPZ in the gastrointestinal tract [137]. The DPZ-HA-TFS was optimized with a 2^4^ factorial design. IN administration of the optimized DPZ-HA-TFS in rats resulted in higher C_max_ (4.12-fold), AUC (3.98-fold), t_1/2_ (1.89-fold), and MRT (1.82-fold) in the rat brain than the IV DPZ. Similarly, quercetin, a nutraceutical compound that can protect the brain against oxidative stress-induced neurodegeneration, was loaded into transferosomes in situ gel for nose-to-brain delivery [138]. The quercetin-loaded transferosomes in situ gel could improve the drug permeation ex vivo and drug accumulation in the rat brain in vivo. 

Niosomes are vesicles produced from non-ionic surfactants, which have a similar structure to liposomes [139]. Natural phospholipids in liposomes are replaced by synthetic surfactants in niosomes. These vesicles are less costly and exhibit improved stability compared to liposomes. Their low toxicity and enhanced stability make them a promising alternative to liposomes [140]. Niosomes have been employed for nose-to-brain delivery. For example, the niosomal in situ gels of citicoline (CTC) were developed for efficient brain delivery of CTC via the IN route [141]. CTC is a psychostimulant and neuroprotective drug used for the management of epilepsy. Its high hydrophilicity and hepatic uptake hinder the drug from passing the BBB, resulting in low brain bioavailability. The pharmacodynamics study in pentylenetetrazole seizure-induced rats revealed that a low dose of CTC-niosomal in situ gel had a powerful protective effect with delayed latency at the start of convulsions. Similarly, niosomal in situ gels of methotrexate (MTX) prepared with CS and Poloxamer 407 exhibited higher brain-to-plasma concentration ratios than other formulations [142]. The brain-to-plasma concentration ratios for MTX–niosomal gel, MTX niosomes, MTX gel, and free MTX were 7, 4, 1.55, and 0.35, respectively.

SLNs and NLCs are advanced nanotechnology-based drug delivery systems that have garnered significant interest due to their biocompatibility and ability to enhance the bioavailability of drugs [143,144]. SLNs are composed of biocompatible lipids that remain solid at room and body temperatures. NLCs, often regarded as second-generation SLNs, incorporate solid and liquid lipids, allowing for improved drug loading capacity and stability [145]. SLNs and NLCs are considered alternatives to other nanocarriers like liposomes or polymeric nanoparticles, offering better entrapment efficiency, particularly for hydrophobic drugs, and making them ideal for clinical applications. SLNs and NLCs can be produced using solvent emulsification, injection, high-pressure homogenization, phase inversion temperature, membrane contactor, and coacervation methods [146,147].

SLNs and NLCs have been used for the nose-to-brain delivery of various drugs. For example, sumatriptan-loaded NLCs were optimized using the D-optimal design [148]. In the pharmacokinetics studies using rats, the DTE% and DTP% of the IN administered NLCs were 258% and 61.2%, respectively, suggesting the desirable entrance of sumatriptan into the brain. Asiatic acid (AA), a compound with neuroprotective potential for preventing and treating AD, was encapsulated into SLNs prepared from rice bran wax, Tween 80, and soybean lecithin for nose-to-brain delivery [149]. The SLNs exhibited higher brain distribution after IN administration in mice than IV administration. In another study, IN administration of AA-loaded SLNs enhanced learning and memory abilities in rats when testing with Morris water maze and novel object recognition tests. The IN AA-loaded SLNs also inhibited tau hyperphosphorylation, glial activation, and lipid peroxidation in AD rats induced by Aβ_1-42_, indicating their potential in treating the early stages of AD [150].

NLCs loaded with paliperidone were developed for nose-to-brain targeting [151]. The NLCs exhibited three-fold higher ex vivo permeation compared to the pure drug. The pharmacokinetics study in rats showed higher drug distribution to the brain after IN administration of the NLCs. Previously, a teriflunomide (TFM)-loaded NLC carbopol-gellan gum in situ gel was developed to improve the brain delivery of the drug [152]. The TFM-NLC gel showed higher ex vivo nasal permeation than the TFM-NLCs. The pharmacokinetics study in mice revealed that the IN TFM-NLC gel had higher C_max_ and AUC in the brain compared with the IN TFM-NLCs and IV TFM-NLCs. In another study, SLNs loaded with sumatriptan were developed and characterized in vitro. However, no in vivo data demonstrated the nose-to-brain delivery of the SLNs [145]. Rizatriptan, a drug used to treat the symptoms of migraine headaches, was loaded into a lipid NP nasal spray (LNP-NS) to improve the drug delivery to the brain [153]. The optimized formulation demonstrated a significantly higher olfactory deposition fraction and an accelerated onset of action (5 min) in rats following nasal spray. The C_max_ and AUC for LNP-NS were higher than those for oral tablets and IV groups. The LNP-NS also maintained a prolonged and elevated drug concentration in the brain for 120 min. In a nitroglycerin-induced acute migraine rat model, the abnormal behavior duration of the LNP-NS group was reduced by 32.04% [153]. Similarly, IN fluoxetine-loaded NLCs reduced depressive and anxiety-like behaviors of mice in the marble-burying test and forced swimming test [154].

Table 3 summarizes the significant features of lipid-based nanocarriers for nose-to-brain delivery found in these studies.

### 5.4. Other Nanocarrier Systems

In addition, other nanocarriers have been used for the nose-to-brain delivery of drugs. Polymeric lipid hybrid NPs (PLNs) are core–shell NPs consisting of a polymeric core and a lipid shell. PLNs are physically stable and biocompatible [147]. PLNs can be produced from a mixture of different polymers and lipids, making them suitable for encapsulating various bioactive molecules in their core and shell [155]. In a previous study, rivastigmine (RIV), a drug widely used in AD therapy, was combined with docosahexaenoic acid (DHA) to form an RIV:DHA ion pair complex [156]. The complex was loaded into cationic and anionic PLNs by using different lipids. PLGA was used as a hydrophobic polymer. The cationic and anionic PLNs were incorporated into the hydrogel of Poloxamer 407 and Poloxamer 188, which exhibited higher ex vivo nasal permeation than the free drug gel (4.07- and 3.18-fold, respectively). The mucociliary time in the rat nasal cavity for the cationic and anionic PLN gels was 3.18- and 2-fold higher than the free drug gel. In the pharmacokinetics study in rats, cationic and anionic PLN gels increased C_max_ in the brain by 2.37- and 1.99-fold, MRT by 9.26- and 5.63-fold, and AUC_brain_ by 7.67- and 5.18-fold, respectively, as compared with the IN free drug gel. Furthermore, the DTE% values were different in order of free RIV gel (281.3%) < anionic PLN gel (672.3%) < cationic PLN gel (792.5%). Similarly, the DTP% values were in order of free RIV gel (64.4% anionic PLN gel (85.1%) < cationic PLN gel (87.4%) [156]. These values confirmed the efficient brain targeting of IN PLN gels compared to the free drug gel.

In another study, two types of NPs, including mesoporous silica NPs and magnetic mesoporous silica NPs, were developed to deliver ponatinib, a drug used to treat glioblastoma, to the brain following IN administration [157]. In vitro BBB permeability of ponatinib was tested in MDCK-MDR1 monolayers, which showed the permeability in the order of free drug < mesoporous silica NPs < magnetic mesoporous silica NPs. Forty-eight hours after IN administration, mesoporous silica NPs and magnetic mesoporous silica NPs exhibited 8.9- and 4.1-fold higher ponatinib concentrations in rat brains than the free drug.

Nanosuspensions were also employed for nose-to-brain delivery [158]. Clozapine (CLZ), a drug used to treat schizophrenia, has low brain distribution following oral administration due to its low solubility, poor dissolution rate, degradation in the gastrointestinal tract, and high hepatic first-pass metabolism. In a previous study, CLZ nanosuspensions were prepared using high-speed homogenization with TPGS as a stabilizer [158]. Following IN administration, the optimal CLZ nanosuspensions exhibited a 3.56-fold increase in drug concentration in rat brains with a 528-fold lower drug dose compared with the oral administration of a conventional CLZ suspension. 

Table 4 summarizes the major features of other types of nanocarriers for nose-to-brain delivery in these studies.

### 5.5. Material Used for Nanocarriers

Various materials are used to develop nanocarriers for nose-to-brain Poloxamers 407 and 188, with amphiphilic properties that are widely used to form micelles [98,108]. They are also typical polymers to produce thermosensitive in situ gels that transition to a gel state at body temperature, providing prolonged mucosal retention for other nanocarriers [101,116]. Gelling agents like Carbopol 940, Carbopol 974P, and gellan gum are utilized to formulate hydrogels, which serve as matrices to load nanocarriers, enhancing mucoadhesion and controlled release at the nasal cavity [105]. Copolymers such as PEG-PLA, PEG-PLGA, and PCL-PVA-PEG are employed in micelle preparation, offering tunable degradation and drug release profiles suited for CNS delivery [99,100,107,109]. For polymeric NPs, materials like PLGA, PEG-PLGA, and CS are favored for their biocompatibility and ability to penetrate mucus [114,123]. CS also provides mucoadhesive properties that boost paracellular transport [122]. Materials for the fabrication of liposomes include phosphatidylcholine, lecithin, and cholesterol [132,133]. Various lipids are used for the preparation of emulsions, SLNs, and NLCs, such as stearic acid, glyceryl monostearate, glyceryl di-behenate, glyceryl mono-linoleate, Gelucire 44/14, Capmul MCM, and oleic acid [125,148,151].

## 6. Authors’ Perspectives

Some clinical trials have been carried out to evaluate various drugs for different CNS diseases. For instance, diazepam (NRL-1) was used in a Phase 3 trial for epilepsy (NCT02721069). Dihydroergotamine with advanced POD devices for migraine management completed a Phase I study for PK and safety (NCT03874832) and a Phase 3 trial for safety and tolerability (NCT03557333). Esketamine has been extensively studied for major depressive disorder, with a Phase 3 study comparing its nasal spray to quetiapine extended release (NCT04338321), another Phase 3 for efficacy and safety (NCT03039192), and a Phase 2 pediatric study for efficacy and safety of three fixed doses with midazolam (NCT03185819). Lidocaine was used in a Phase 1 trial for pediatric migraine (NCT03806595), and zolmitriptan (Zomig^®^) was used in a Phase 3 study for young migraine patients (NCT03275922). Olanzapine, delivered via a POD device for schizophrenia and bipolar disorder, was used in a Phase 1 safety study (NCT03624322). Zavegepant was used in a Phase 3 trial for migraine (NCT04571060). These trials collectively underscore the advancing role of intranasal delivery in achieving rapid, targeted brain therapies [45]. The clinical landscape of nose-to-brain drug delivery has advanced significantly, as evidenced by a range of FDA-approved intranasal products targeting central nervous system disorders and other conditions through rapid and direct brain access. For instance, butorphanol, available as a metered spray, serves as an opioid narcotic pain reliever, while diazepam (Valtoco^®^) addresses stereotypic episodes with a spray formulation. Migraine treatment is well-represented with dihydroergotamine in metered sprays like Migranal^®^ and Trudhesa^®^, alongside sumatriptan in spray forms (Imitrex^®^ and Tosymra^®^) and as a powder spray (Onzetra^®^ Xsail^®^). Esketamine, delivered via Spravato^®^, targets major depressive disorder, showcasing the route’s potential for psychiatric applications. Additionally, naloxone (Narcan^®^) provides a life-saving metered spray for opioid overdose reversal. These approved therapies, spanning sprays and powders, highlight the clinical maturity of nose-to-brain delivery, offering fast-acting, non-invasive options for acute and chronic conditions, with ongoing innovation in device and formulation design driving further therapeutic possibilities [45].

Various nanocarriers have been developed and evaluated for the nose-to-brain delivery of various drugs. Findings from recent studies demonstrate that nanocarriers, including micelles, NEs, liposomes, polymeric NPs, SLNs, NLCs, and PLNs, can effectively deliver drugs to the brain via direct nose-to-brain routes following IN administration. Considering the pharmacokinetic parameters such as AUC_brain_, DTE%, and DTP% in previous studies (Table 1, Table 2, Table 3 and Table 4), we identify several promising drug-loaded nanocarriers for nose-to-brain delivery. Among micelle-based formulations, the most promising ones are clozapine micelles prepared with Tetronic^®^ 904, Tetronic^®^ 701, Synperonic^®^ PE/F127 (DTE = 396.5%) [102], olanzapine micelles prepared with Poloxamer 407, Pluronic P123, and TPGS (DTE = 535.9%, DTP = 81.3%) [106], and the hydrogel of lurasidone-loaded mixed micelles (DTE = 394%, DTP = 74%) [105]. Typical polymeric NPs are PBD-LCN-ISG (PBD lecithin-CS NPs loaded in a methylcellulose thermo-responsive in situ gel) for Parkinson’s disease with increased AUC_brain_ (6.4-fold) and DTP = 56% [119] and DXH PLGA-CS NPs for treating major depressive disorder with improved C_max (brain)_ (3.33-fold) and AUC_brain_ (3.57-fold) [122]. Different NEs are effective in enhancing nose-to-brain delivery, such as naringenin-loaded NEs in situ gel with DTE = 1224% and DTP = 99.5% [125], aripiprazole-nanoemulgels with DTE = 974% and DTP = 89.73% [126], and tetrabenazine NEs with increased C_max (brain)_ (4-fold) and AUC_brain_ (6.07-fold), DTE = 1666%, and DTP = 94% [128]. Other lipid-based nanocarriers are also potential, such as sumatriptan-loaded NLCs (DTE = 258%, DTP = 61.2%) [148] and donepezil transfersomes with increased C_max (brain)_ (4.12-fold) and AUC_brain_ (3.98-fold) [137].

However, IN drug delivery presents several challenges that must be addressed during formulation development to ensure effective and safe administration. A key limitation is the restricted volume administered per nostril, typically ranging between 25 and 200 µL [32]. This small volume can be insufficient for drugs that require higher doses, making it difficult to achieve therapeutic concentrations via this route. Moreover, the nasal cavity has a limited surface area for absorption compared to larger organs like the gastrointestinal tract, which can further limit the amount of drug absorbed. The presence of the mucociliary clearance system, which serves as a natural defense mechanism, quickly clears foreign substances from the nasal cavity, reducing the time the drug remains in contact with the mucosa to less than 30 min [15]. This rapid clearance can significantly restrict the absorption window, limiting the bioavailability of the drug. Additionally, certain drugs face degradation in the nasal cavity due to the presence of proteolytic enzymes, such as aminopeptidases and esterases [13,14]. This enzymatic degradation can reduce the effectiveness of peptides, proteins, and other sensitive molecules. Hydrophilic drugs, in particular, often face challenges with poor permeability across the nasal epithelium, necessitating permeation enhancers or specialized formulations to improve their transport. Furthermore, the nasal mucosa is highly sensitive, and some formulations may cause irritation or inflammation, particularly if they disrupt the epithelial barrier [159]. This risk underscores the importance of optimizing the formulation’s pH, osmolality, and viscosity to ensure compatibility with the nasal environment and minimize adverse effects. Additional challenges arise from variability in drug absorption due to nasal conditions, such as congestion, infections, or other nasal medications. These factors can significantly alter the permeability and clearance rates, leading to inconsistent drug delivery outcomes. As such, the development of IN formulations must carefully consider factors like pH stability, residence time, enzyme resistance, and individual patient conditions to optimize both efficacy and safety. Overcoming these challenges may involve the use of mucoadhesive agents to prolong residence time [119] and permeation enhancers to improve absorption [126]. Nanocarriers can also enhance the drug’s solubility and permeation. Long-term safety and potential neurotoxicity of nanocarriers in the brain are also critical issues that must be addressed. Some studies have reported the low toxicity of these nanocarriers on mucosal tissues [145]. However, brain toxicity has not been thoroughly evaluated. Therefore, future research should focus on comprehensive safety evaluations.

The development of nanocarriers for nose-to-brain drug delivery has increased in the past decade. The recent advances in the studies discussed above have demonstrated the efficacy and potential of nanocarriers for nose-to-brain delivery. We expect that they will enhance the management of a wide range of CNS diseases in the near future.

## Figures and Tables

**Table 1:** Major features of micelle-based formulations for nose-to-brain delivery.

Drug	Components	Outcome	Ref.
Risperidone	Poloxamer 407 and 188	Size: 118 nm, PDI: 0.315 Increased permeation across a cellulose membrane	[98]
Dexamethasone	PCL-PVAc-PEG, TPGS	Size: 90 nm, PDI: 0.216, ZP: –21.1 mV, EE: 93.4% Increased aqueous solubility (14-fold), enhanced permeability (in vitro passive diffusion test and parallel artificial membrane permeability assay)	[99]
Baicalein	PEG-PLA	Size: 25 nm, PDI: 0.239, ZP: –7.3 mV, EE: 70%, DL: 1.39% Reduced drug toxicity, reduced inflammatory factors TNF-α and IL-6 Increased BA (inhalation, mice): 5.09-fold (plasma) and 1.50-fold (brain)	[100]
Rotigotine	mPEG-PLGA; poloxamer 407 and 188 (gelling agents)	Size: 88 nm, PDI: 0.237, EE: 94%, DL: 19.9% Sustained release, no obvious side effects on the nasal cilia and rat nasal mucosa Increased drug distribution in olfactory bulb (276.6%), cerebrum (170.5%), cerebellum (166.5%), and striatum (184.4%).	[101]
Clozapine	Tetronic^®^ 904, Tetronic^®^ 701, Synperonic^®^ PE/F127	Size: 217 nm, PDI: 0.24 Increased nasal permeation ex vivo (5-fold) Increased brain distribution in mice (vs. IV) with DTE = 396.5%	[102]
Clozapine	SPC, SDC	Size: 12.2 nm, PDI: 0.24, ZP: –38 mV, EE: 93%, DL: 6.47% Higher ex vivo permeation (3-fold) Rapid onset (15 min) and higher brain bioavailability (vs. IV)	[103]
Ibudilast	Surfactin, polydopamine (for coating)	Size: 175 nm, PDI: 0.3, ZP: –41 mV, EE: 87.6% Increased drug distribution to mouse brain Positive outcome in treating multiple sclerosis (anti-inflammation and neuroprotection)	[104]
Lurasidone	Gelucire 44/14, Poloxamer 407; carbopol 940 (gelling agent)	Size: 175 nm, EE: 97.8% Increased nasal permeation ex vivo (1.3-fold) Increased brain distribution in rat (vs. IV) with DTE = 394% and DTP = 74%	[105]
Olanzapine	Poloxamer 407, Pluronic P123, TPGS	Size: 39 nm, ZP: –15 mV, EE: 82% Increased brain distribution in rats with DTE = 535.9% and DTP = 81.3% Improved anti-schizophrenia-related deficits via the paw test and open field test Safe as indicated by histopathological examination	[106]
Meloxicam	PCL-PVAc-PEG	Size: 101 nm, PDI: 0.149, EE: 94%, ZP: –25.2 mV, EE: 89% Increase permeation across the culture model of the nasal mucosa barrier (5-fold) AUC_brain_ is only 0.65% of AUC_plasma_	[107,109]
Clozapine	Polysorbate 20 & 80, Poloxamer 407	Size: 17–20 nm, PDI: 0.3, ZP: –2.7 mV Reduced permeation across nasal mucosal tissues No in vivo data	[108]

PEG-PLA, poly(ethylene glycol)-block-poly(D, L-lactide); mPEG-PLGA, methoxy-poly(ethylene glycol)-block-poly(lactic-co-glycolic acid); PCL-PVAc-PEG, polyvinyl caprolactam–polyvinyl acetate–polyethylene glycol graft co-polymer; TPGS, D-α-tocopherol polyethylene glycol 1000 succinate; IV: intravenous; DTE, drug targeting efficiency; SPC, soya phosphatidyl choline; SDC, sodium deoxycholate; BA: bioavailability.

**Table 2:** Major features of polymeric NPs for nose-to-brain delivery.

Drug	Components	Outcome	Ref.
Edaravone	PLGA, PLGA-PEG	Size: 90 nm, PDI: 0.214, ZP: –11.9 mV, EE: 20.58%, DL: 3.02% Reduced oxidative stress toxicity in mouse microglial cell line BV-2 Increased brain distribution in mice (vs. IV free drug and IV NPs)	[114]
Meloxicam	Human serum albumin; Poloxamer 407 (gelling agent)	Size: 176 nm, PDI: 0.205, ZP: –7.9 mV, EE: 81.64%, DL: 1.09% Increased drug permeation ex vivo	[115]
Baicalin	PEG-PLGA, RVG29 peptide	Size: 89–130 nm, PDI: 0.1–0.3 Reduced neurological dysfunction and oxidative stress in rats with ischemic brain injury	[113]
Vinpocetine	CS; Poloxamer 407 and Poloxamer 188 (gelling agent)	Size: 130.6 nm, PDI: 0.125, ZP: 40.81 mV, EE: 97.56% Increased C_max_ (2.2-fold) and AUC (1.7-fold) in rat brain (vs. oral tablets)	[116]
Astragaloside IV	CS, β-asarone	Size: 118 nm, PDI: 0.253, ZP: 22.7 mV, DL: 0.14% Increased in vitro uptake (1.52-fold) and brain delivery (2.49-fold) (β-asarone-CS-NP vs. CS-NP). Reduced behavioral scores, decreased weight loss, suppressed inflammatory infiltration and astrocyte/microglial activation, reduced demyelination, and increased remyelination on an EAE mouse model.	[117]
Piribedil	CS, lecithin, methylcellulose in situ gel	Size: 147 nm, PDI: 0.29, ZP: 18.1 mV, EE: 53.5%, DL: 12.1% Increased brain bioavailability (IN NP gel > IN NP suspension > IN free drug suspension) with DTE = 228% and DTP = 56% for IN NP gel and DTE = 140% and DTP = 29% for IN NP suspension	[119]
Paclitaxel	PLGA, arginyl-glycyl-aspartic tripeptide	Size: 197 nm, PDI: 0.192, DL: 2.8% Increased brain accumulation in rats Reduced 72% tumor volume in rats implanted with C6 glioblastoma cells Reduced 75% tumor volume in mice implanted with U87MG glioblastoma cells	[120]
Clonidine	PLGA, transferrin	Size: 200 nm, PDI: 0.291, ZP: –17.4 mV, EE: 86.2%, DL: 7.8% Increased Neuro-2a cell uptake (97% vs. 82%) Increased drug concentration in mouse brain (2.4-fold vs. IN free drug) Improved behavioral responses	[121]
Paroxetine	CS, PLGA	Size: 182 nm, ZP: 36.3 mV, EE: 87.5%, DL: 13.4% Increased drug concentration in mouse brain Improved behavioral responses in forced swimming test and locomotor activity test	[123]
Duloxetine	PLGA, CS, PVA	Size: 122 nm, EE: 66.95% Increased ex vivo permeation (4-fold vs. drug solution) Improved behavior via the force-swimming test, tail suspension test, sucrose preference test, open field test, and novelty suppressed feeding Increased C_max_ (3.33-fold), AUC (3.57-fold), t_1/2_ (1.76-fold), and MRT (1.43-fold) in rat brain vs. oral free drug	[122]

**Table 3:** Major features of lipid-based nanocarriers for nose-to-brain delivery.

Nanocarrier	Drug	Components	Outcome	Ref.
NEs	Naringenin	Capmul MCM, Tween-80, PEG-400; Poloxamer 407 (gelling agent); CS (mucoadhesive agent)	Size: 98 nm, PDI: 0.386 Increased brain bioavailability with DTE = 1224% and DTP = 99.5% Improved locomotor activity and grip strength in rats Increased antioxidant enzyme levels (superoxide dismutase, catalase, glutathione peroxidase, and glutathione reductase)	[125]
NEs	Aripiprazole	Capmul PG-8, TPGS, Transcutol-HP; Carbopol 971 (gelling agent)	Size: 140 nm, PDI: 0.401, ZP: −16.87 mV Increased ex vivo permeation (~2-fold vs. drug solution) Increased C_max_ and AUC in rat brain. DTE = 974%, DTP = 89.73% Improved behaviors in rats (catalepsy, induced locomotor activity, and paw test)	[126]
NEs	Topiramate	Capmul MCM C8, Tween 20, Carbitol	Size: 4.73 nm, PDI: 0.206, ZP: 10.74 mV Decreased seizure duration (vs. oral NEs, IN free drug, and oral free drug) in rats Increased drug distribution to rat brains (vs. oral NEs, IN free drug, and oral dree drug)	[127]
NEs	Tetrabenazine	Capmul MCM, Tween 80, Transcutol P	Size: 106.8 nm, PDI: 0.198, ZP: –9.63 mV Increased ex vivo permeation (1.68-fold vs. free drug) Increased C_max_ (4-fold) and AUC (6.07-fold) in rat brains vs. IV drug solution DTE = 1666%, DTP = 94%	[128]
Liposomes	Imatinib mesylate	Egg PC, cholesterol, and cardiolipin	Size: 102 nm, PDI: 0.28, ZP: −23 mV Increased AUC in rat brains (7-fold) vs. oral and IN-free drugs	[132]
Liposomes	Hydroxy-α-sanshool	Soybean lecithin, cholesterol	Size: 182 nm, PDI: 0.207, ZP: −54 mV, EE: 73% Increased mouse plasma (1.7-fold) and brain (2.1-fold) bioavailability (vs. free drug)	[133]
Transfersomes	Donepezil	PC, HA, Tween 80	Size: 227.5 nm, EE: 75.8% Increased C_max_ (4.12-fold), AUC (3.98-fold), t_1/2_ (1.89-fold), and MRT (1.82-fold) in rat brain vs. IV free drug	[137]
Transfersomes	Quercetin	Lecithin, sodium deoxycholate; Carbopol 971P, Poloxamer 188, Poloxamer 407 (gelling agents)	Size: 171 nm, ZP: −32.6 mV, EE: 78.2% Increased ex vivo permeation (2-fold vs. free drug gel) Increased drug accumulation in rat brain	[138]
Niosomes	Citicoline	Cholesterol, Span-60; Pluronic F-127, Pluronic F-68, HPMC K15M (gelling agents)	Size: 209 nm, ZP: −55.3 mV, EE: 36.65% Improved anticonvulsant activity against rats with pentylenetetrazole seizure induction (vs. oral free drug)	[141]
Niosomes	Methotrexate	Cholesterol, Span-60; Poloxamer 407, CS (gelling agents)	Size: 130.5 nm, PDI: 0.536, ZP: –38.5 mV, EE: 91.39% Increased brain-to-plasma concentration ratio (free drug < drug-loaded gel < drug-loaded niosomes < drug-loaded niosomal gel)	[142]
NLCs	Sumatriptan	Stearic acid, cholesterol, triolein, Brij 35	Size: 101 nm, PDI: 0.27, EE: 91% Increased bain bioavailability with DTE = 258% and DTP = 61.2% in rats	[148]
SLNs	Asiatic acid	Rice bran wax, Tween 80, soybean lecithin	Size: 197 nm, PDI: 0.25, ZP: –31.6 mV, EE: 99.9% Increased brain distribution in mice (vs. IN solution and IV SLNs)	[149]
SLNs	Asiatic acid	N/A	Improved spatial memory dysfunction, recognition memory impairment, reduced tau hyperphosphorylation, inhibited glial activation and lipid peroxidation in Aβ_1-42_-injected rats.	[150]
NLCs	Paliperidone	Glyceryl monostearate, oleic acid, Tween 80	Size: 129 nm, PDI: 0.304, ZP: −7.61 mV, EE: 58.16% Increased ex vivo drug permeation (3-fold vs. free drug) Increased drug delivery to rat brain	[151]
NLCs	Teriflunomide	Glyceryl di-behenate, glyceryl mono-linoleate, Gelucire 44/14; Carbopol 974P and gellan gum (gelling agents)	Size: 117.8 nm, PDI: 0.56, ZP: −21.86 mV, EE: 81.16% Increased permeability coefficient (1.53-fold vs. NPs) Increased C_max_ (2-fold) and AUC (1.34-fold) in mouse brain (vs. IV and IN NPs)	[152]
SLNs	Sumatriptan	Soya lecithin, CS, tripalmitin	Size: 133 nm, ZP: –17.7 mV, EE: 75.4% Safety via histopathological evaluation of mucosal tissue	[145]
LNPs	Rizatriptan	EPC, cholesterol	Size: ~100 nm, PDI: ~0.25, ZP: ~ –23 mV Quick onset (5 min), higher C_max_ and AUC in rats (vs. oral tablets and IV) Prolonged drug concentration in the brain for 120 min Reduced abnormal behavior duration by 32.04%.	[153]
LNPs	Rizatriptan	EPC, cholesterol, borneol	Size: ~120 nm, PDI: ~0.2, ZP: ~ –20 mV Increased drug absorption in nasal mucosa (1.37-fold), AUC_brain_ (1.23-fold) vs. non-modified LNPs. Reduced abnormal behavior duration by 56.64%. Alleviated symptoms of neuroinflammation-induced hyperalgesia	[153]
NLCs	Fluoxetine	Precirol™ ATO 5, Lauroglycol™ 90, Tween^®^ 80	Size: 154 nm, PDI: 0.514, ZP: 19.7 mV, EE: 74%, DL: 12.9% Reduced depressive and anxiety-like behaviors of mice in the marble-burying test and forced swimming test	[154]
SLNs	Asiatic acid	Rice bran wax, Tween 80, soybean lecithin	Size: 197 nm, PDI: 0.25, ZP: –31.6 mV, EE: 99.9% Increased brain distribution in mice (vs. IN solution and IV SLNs)	[149]

NEs: nanoemulsions; LNPs: lipid nanoparticles.

**Table 4:** Major features of other nanocarriers for nose-to-brain delivery.

Nanocarrier	Drug	Components	Outcome	Ref.
PLNs	Rivastigmine-DHA	PLGA, stearyl amine, Miglyol 812, Span-80; Poloxamer 407 and 188 (gelling agents)	Size: 132 nm, PDI: 0.284, ZP: 36.4 mV, EE: 83.6% Increased ex vivo nasal permeation (4.07-fold vs. free drug gel) Increased mucociliary time (2-fold vs. free drug gel) Increased C_max_ (2.37-fold), MRT (9.26-fold), and AUC_brain_ (7.67-fold) (vs. IN free drug gel) DTE = 792.5%, DTP = 87.4%	[156]
PLNs	Rivastigmine-DHA	PLGA, glyceryl monostearate, PEG-32-stearate; Poloxamer 407 and 188 (gelling agents)	Size: 160 nm, PDI: 0.254, ZP: –39.3 mV, EE: 88.2% Increased ex vivo nasal permeation (3.18-fold vs. free drug gel) Increased mucociliary time (1.4-fold vs. free drug gel) Increased C_max_ (1.99-fold), MRT (5.63-fold), and AUC_brain_ (5.18-fold) (vs. IN free drug gel) DTE = 672.3%, DTP = 85.1%	[156]
Silica NPs	Ponatinib	Cetyltrimethylammonium bromide, diethanolamine, tetraethylorthosilicate	Increased in vitro BBB permeability (vs. free drug) Increased drug concentration in rat brains at 48 h (vs. free drug)	[157]
Nanosuspension	Clozapine	TPGS, PVP K-30	Size: 281 nm Increased drug concentration in rat brains (3.56-fold with a 528-fold lower dose vs. conventional suspension)	[158]

PLNs: polymer lipid hybrid NPs.

## Data Availability

Not applicable.
